# DNArepairK: An Interactive Database for Exploring the Impact of Anticancer Drugs onto the Dynamics of DNA Repair Proteins

**DOI:** 10.3390/biomedicines9091238

**Published:** 2021-09-16

**Authors:** Yordan Babukov, Radoslav Aleksandrov, Aneliya Ivanova, Aleksandar Atemin, Stoyno Stoynov

**Affiliations:** 1 Faculty of Mathematics and Informatics, Sofia University, St. Kliment Ohridski, 5 James Bourchier Blvd., 1164 Sofia, Bulgaria; yordanbabukov@gmail.com; 2 Laboratory of Genomic Stability, Institute of Molecular Biology, Bulgarian Academy of Sciences, Acad. G. Bonchev Str. Bl.21, 1113 Sofia, Bulgaria; raleksandrov@bio21.bas.bg (R.A.); anivanova@bio21.bas.bg (A.I.); atemin@bio21.bas.bg (A.A.)

**Keywords:** DNA damage response, DNA repair protein kinetics, live cell imaging, laser micro-irradiation, PARP1/2 inhibitors, talazoparib, anticancer drug development, CRC mathematical modeling, DNArepairK database

## Abstract

Cells are constantly exposed to numerous mutagens that produce diverse types of DNA lesions. Eukaryotic cells have evolved an impressive array of DNA repair mechanisms that are able to detect and repair these lesions, thus preventing genomic instability. The DNA repair process is subjected to precise spatiotemporal coordination, and repair proteins are recruited to lesions in an orderly fashion, depending on their function. Here, we present DNArepairK, a unique open-access database that contains the kinetics of recruitment and removal of 70 fluorescently tagged DNA repair proteins to complex DNA damage sites in living HeLa Kyoto cells. An interactive graphical representation of the data complemented with live cell imaging movies facilitates straightforward comparisons between the dynamics of proteins contributing to different DNA repair pathways. Notably, most of the proteins included in DNArepairK are represented by their kinetics in both nontreated and PARP1/2 inhibitor-treated (talazoparib) cells, thereby providing an unprecedented overview of the effects of anticancer drugs on the regular dynamics of the DNA damage response. We believe that the exclusive dataset available in DNArepairK will be of value to scientists exploring the DNA damage response but, also, to inform and guide the development and evaluation of novel DNA repair-targeting anticancer drugs.

## 1. Introduction

DNA molecules carry all the genetic information that governs the development and functions of human cells. It has been shown that the DNA in every cell suffers from numerous lesions caused by both internal and external mutagenic factors [1]. Nowadays, it is estimated that every single nucleated cell in the human body experiences between 10,000 and 100,000 mutations in its DNA on a daily basis. Those mutations, if left unaccounted, could lead to cell transformation, cell cycle arrest, cell senescence, or cell death [2]. The accumulation of mutations in DNA have been associated with many degenerative diseases and is often cited as one of the main causes behind aging [3]. Therefore, eukaryotic cells have evolved a remarkably complex protein machinery termed collectively as the DNA damage response (DDR), which is responsible for DNA damage detection and repair. DDR comprises more than several hundred different cellular proteins and protein complexes organized into damage-specific DNA repair pathways that deal with different types of DNA lesions [4]. The repair of DNA is a highly ordered process, both in space and time, during which different DNA repair proteins are recruited to the damaged chromatin in a chronological order, depending on their function [5]. Altering the order of recruitment or dissociation of DNA repair proteins due to genetic mutations or pharmacological perturbations may severely compromise its efficiency, leading to excessive genomic instability and cell death [6].

During the past several decades, a great amount of knowledge has been gained concerning the functions, the biochemical properties, and the interactions between hundreds of different DNA repair proteins [7]. However, the temporal dynamics and coordination of the DNA damage response is still rudimentary characterized. Although the kinetics of many DNA repair proteins have been reported in the literature, these data are not readily comparable, because they have been obtained in different cell lines, by using different methods for DNA damage infliction, non-standardized analysis approaches, etc. [8]. Recently, we reported the high-resolution kinetics of recruitment and removal of 70 different fluorescently labeled DNA repair proteins at UV laser-induced DNA damage sites in living HeLa Kyoto cell lines obtained by live cell imaging in uniform experimental conditions [9]. In addition, we employed a mathematical model, termed the Consecutive Reactions Chain (CRC) model, to further investigate and characterize the kinetics of all studied proteins. Our study put most of the events occurring in the course of DNA repair in a precise time frame and uncovered unexpected aspects concerning the temporal coordination of DDR in living cells. Moreover, we systematically investigated the impact of the PARP1/2 inhibitor (PARPi) talazoparib (BMN673) onto the dynamics of the DNA repair process [10]. We uncovered that talazoparib treatment profoundly changed the kinetics of recruitment and/or removal of multiple DDR proteins at the sites of DNA lesions compared to non-treated cells. Such an effect could cause a severely reduced efficiency of the DDR due to the compromised order of recruitment and coordination between different DNA repair proteins benefiting the killing of cancer cells. These results rationalize our tactics as a viable approach for the discovery and evaluation of novel DDR targeting anticancer drugs in the preclinical setting. However, all these experimental data and mathematical models are currently not easily accessible and readily comparable, which hinders the exploration of this vast dataset. Therefore, the creation of an interactive and open-access platform containing all the data that we have previously obtained would greatly facilitate the investigation of this unique dataset and benefit many scientists working in the field of the DNA damage response.

Here, we present DNArepairK, an interactive and user-friendly database that collects and provides access to all the experimental kinetics data that we have obtained [9], thereby facilitating the exploration of the highly complex temporal dynamics of the DNA damage response. All raw kinetic curves are accompanied by their respective CRC models and kinetic constants used to quantitatively describe the obtained live cell imaging data for every single protein. One of the main advantages of DNArepairK is the possibility to visualize and compare the kinetics of multiple DNA repair proteins obtained in the same experimental conditions, which is a unique feature of the DNArepairK database. For most of the proteins included in DNArepairK (46 out of 70), users are able to investigate and compare their kinetics with and without talazoparib treatment, thereby gaining insights about the effects of PARP1/2 inhibition. Furthermore, the kinetics of every single protein is accompanied by a movie depicting its recruitment and removal in living cells. The database also includes an interactive slideshow of the different steps in the course of DNA repair that is based on the kinetic data presented as part of this database and the current knowledge about the functions and interactions between the included proteins.

## 2. Materials and Methods

### 2.1. Data Acquisition

**Figure 1:**
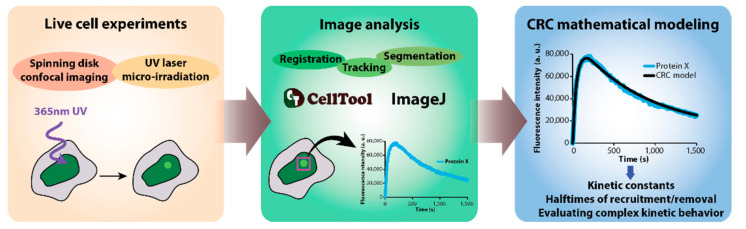
DNArepairK dataset acquisition.

The DNArepairK database includes the kinetics of recruitment of 70 different DNA repair proteins to UV laser-induced complex DNA damage sites in living HeLa Kyoto cell lines. This large dataset was published by Aleksandrov et al. [9], and all aspects concerning data acquisition and image analysis are described in detail there. Shortly, we used HeLa Kyoto cell lines generated by Bacterial Artificial Chromosome (BAC) recombineering, which express fluorescent protein-labeled DNA repair proteins (Figure 1) [11,12]. Since the large size of the BAC transgenes ensures the presence of most, if not all, regulatory elements, the genes for the tagged proteins of interest are under the control of their endogenous promoters and most of their endogenous regulatory sequences while also including all the exons and introns [12]. Notably, these BAC-tagged cell lines have been successfully used for quantitative proteomics, and it has been evaluated and confirmed that BAC-tagged proteins are expressed at near-physiological levels [13,14]. Moreover, the cell cycle and developmental control was demonstrated at full power by Sarov et al. [15], where EGFP-BAC-tagged transcription factors were expressed in specific cells in the course of the development of the nematode *C. elegans*. Therefore, in the cell lines we used to generate this dataset, the expression of all tagged proteins was close to the physiological levels, under precise endogenous cellular control, and the proteins were expressed with all their splice isoforms.

To acquire the precise dynamics of recruitment and removal of the investigated proteins, we used spinning disk confocal microscopy (Andor Revolution system, Andor, Belfast, UK), which is ideally suited to follow processes in living cells for extended periods of time with almost no photobleaching and phototoxicity [9,16]. For image acquisition during all experiments included in DNArepairK, we used a Nikon Eclipse Ti-E microscope (Nikon, Tokyo, Japan) equipped with a Nikon CFI Plan Apo VC 60× water immersion objective with a numerical aperture (NA) of 1.2 (Nikon, Japan). Signal detection was accomplished by a high-sensitivity iXon897 EMCCD camera (Andor, UK). The time intervals between successive time points varied between 0.5 s and 5 s, depending on the kinetics of the particular protein under study. In addition, every time point was acquired in three Z planes, which were later combined using maximum intensity projection to obtain higher-quality images for image analysis [9].

In order to produce DNA lesions in living cells, we employed a 365 nm pulsed UV laser (Micropoint, Andor, UK). The maximum output energy of this micro-irradiation laser system was 150 μJ, which was further attenuated to 70% or 80% (low-power and high-power irradiation, respectively) of this initial value for the live cell imaging experiments. The imaging conditions and micro-irradiation details are provided in the downloadable spreadsheets for every protein included in the DNArepairK database. Micro-irradiation (IR) with this laser system generates complex DNA lesions, i.e., many different types of DNA lesions, in a small and well-defined volume in the nucleus of the cell [9]. Importantly, most natural mutagens, including UV light, ionizing radiation, and many chemicals, including cancer chemotherapeutics, are able to simultaneously induce diverse types of DNA lesions [17,18]. In the course of the time lapse experiments, the cells were maintained at optimal growth conditions in Fluorobrite DMEM (Gibco, rand Island, NY, USA) supplemented with 10% fetal bovine serum (FBS) (Gibco, rand Island, NY, USA), 100 units/mL of penicillin, and 100 μg/mL of streptomycin (Gibco, rand Island, NY, USA) at 37 °C and 5% CO_2_. In order to investigate the systematic effects of the PARP1/2 inhibitor talazoparib (BMN673) onto the dynamics of the repair process, we pretreated the cells with 100 nM talazoparib (Selleckchem, Houston, TX, USA) before obtaining the kinetics of the respective proteins [9].

### 2.2. Image Analysis and Mathematical Modelling

The obtained live cell imaging data included in DNArepairK were analyzed, and the total fluorescence intensity of the recruited DNA repair proteins was measured at DNA damage sites, as previously reported [9]. Briefly, we employed a set of segmentation, registration, and tracking algorithms in ImageJ [19] and CellTool [20] to follow and precisely measure the intensity of UV laser-induced DNA damage foci for the duration of the whole time lapse experiments (Figure 1). The measured total intensity of recruited DNA repair protein molecules is compensated for photobleaching both during micro-irradiation and image acquisition. The number of cells included in the final kinetics for a particular protein varies between 8 and 21.

We employ a specifically devised mathematical model, termed the Consecutive Reactions Chain (CRC) model, in order to comprehensively quantify, compare, and analyze the obtained data [9]. The CRC model approximates the kinetics of recruitment and removal of DDR proteins to a series of consecutive reactions responsible for the recruitment of the protein (characterized by kinetic constants k_1_, k_2_, …, k_n−1_, where “n” is the total number of reactions describing the kinetics of a particular protein), which is followed by a period of time that is spent by the protein at DNA damage sites (detachment delay, τ) and a final dissociation reaction responsible for the detachment of the protein from the lesions (characterized by a single kinetic constant k_n_). The procedure for establishing an appropriate CRC model that adequately describes the experimentally obtained kinetics of a given protein includes fitting a series of equations with an increasing number of “n” to the experimental data until the quality of the fit remains unchanged. It should be noted that such a fitting procedure is capable of pinpointing the minimal number of reactions that are necessary for the recruitment of a particular protein to complex DNA lesions in living cells according to the CRC model, but this does not necessarily mean that “n” shows the actual number of reactions required for its recruitment, i.e., there can be more reactions/events than the CRC model predicts. By employing the CRC model, the kinetic behavior of all the proteins included in DNArepairK was modeled by always using the minimal number of reactions that can precisely describe the dynamics of the respective proteins [9]. The CRC models provide the kinetic constants describing the proteins’ kinetics at DNA damage sites and, thereby, the halftimes of recruitment and removal of the proteins.

## 3. Results

### 3.1. DNArepairK Provides a Unique Dataset for Exploring the Dynamics of the DNA Damage Response

**Figure 2:**
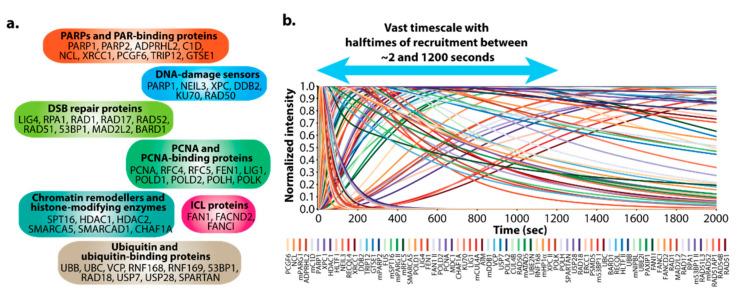
DNArepairK provides a unique dataset for exploring the dynamics of the DNA damage response. (**a**) DNArepairK dataset encompasses proteins implicated in all DNA repair events in human cells. (**b**)The DNArepairK dataset provides an almost complete chronology of the events during complex DNA damage repair.

DNArepairK contains the precise kinetics of 70 different DNA repair proteins at complex DNA lesions generated by UV laser micro-irradiation in living cells [9]. This vast dataset encompasses proteins that participate in all DNA repair pathways and events in human cells—DNA damage sensors (PARP1, PARP2, NEIL3, XPC, DDB2, KU70, RAD50); chromatin remodelers and histone-modifying enzymes (SPT16, HDAC1, HDAC2, SMARCA5); PCNA and PCNA-binding proteins (POLD1, POLD2, POLH, POLK, FEN1, LIG1, CAF1); ubiquitin and ubiquitin-binding proteins (RAD18, RNF168, RNF169, 53BP1); scaffold proteins (XRCC1, MDC1); etc. (Figure 2a). The timescale of recruitment for the proteins included in DNArepairK is formidable, since it comprises proteins with halftimes of recruitment of less than 2 s (PCGF6, NCL) to proteins with halftimes of recruitment of more than 10 min (RPA1, RAD51, RAD52), providing an almost complete temporal map of the order of events during DNA repair (Figure 2b). Due to the complex nature of the lesions we induce, different DNA repair pathways are active within the same chromatin region, and therefore, mechanisms must exist to ensure the correct spatial and temporal coordination of DDR in these conditions. Therefore, the DNArepairK database also provides one-of-a-kind kinetics-based perspective at this intricate coordination and is aimed at simplifying its exploration.

Importantly, all kinetic data that we have included in the database up to this point have been obtained with the same experimental setup (microscope system and micro-irradiation source), same type of cell line, and in the same cellular growth conditions [9]. It is worth noting that the cell lines we employed stably express the proteins of interest at close to physiological levels; thus, our data does not suffer from often-encountered protein overexpression artifacts [21]. Moreover, the cells were never pretreated with DNA damage-sensitizing agents (such as Hoechst), a common practice in micro-irradiation experiments, which could lead to significant deviations in cell physiology and, consequently, in the kinetic behavior of the repair proteins [22]. This makes our data prone to rapid and straightforward comparisons, a unique attribute of the dataset featured in DNArepairK compared to similar databases [23].

### 3.2. DNArepairK Includes CRC Mathematical Models for All Proteins Granting In-Depth Understanding of the Kinetics Data

**Figure 3:**
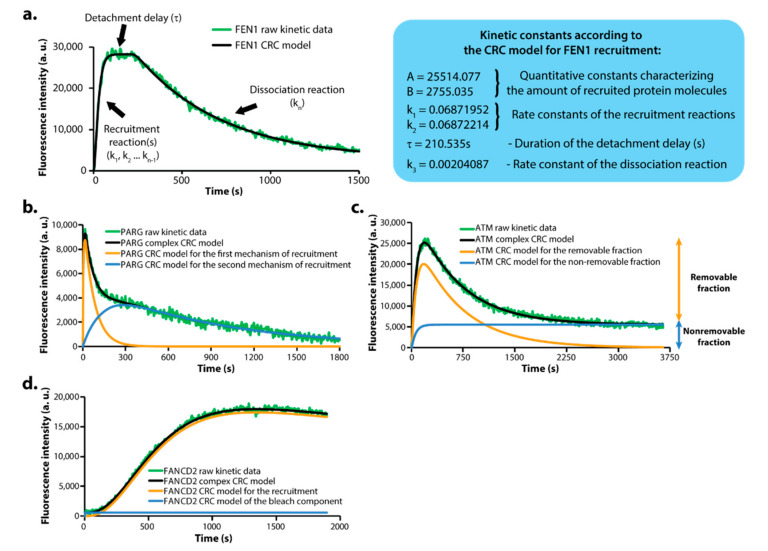
CRC mathematical modelling provides in-depth understanding of the kinetics data. (**a**) General overview of the features of the Consecutive Reactions Chain (CRC) model. (**b**) Modelling the complex kinetic behavior of a protein which is recruited to DNA damage sites by two different mechanisms. The overall CRC model for PARG kinetics is the sum of the two individual CRC models describing the two separate mechanisms for its recruitment. (**c**) Modelling the complex kinetics of a protein which possesses both a removable and a non-removable fraction at DNA damage sites. (**d**) Modelling protein kinetics with not fully compensated bleaching following micro-irradiation. The bleaching component can easily be eliminated by means of the CRC model to facilitate the further kinetics analysis.

The kinetics of recruitment and removal of different DNA repair proteins at complex DNA lesions vary largely. In order to properly quantify these differences and facilitate the comparison between all the proteins, we devised a novel mathematical model termed the Consecutive Reactions Chain (CRC) model [9]. According to this model, the kinetic behavior of a particular DNA repair protein can be approximated as a series of consecutive reactions that are responsible for its recruitment and, consequently, removal from the site of the damage, and every reaction is characterized by a respective rate constant (Figure 3a). Generally, a protein with a shorter halftime of recruitment will be described by a CRC equation that includes a smaller number of recruitment reactions compared to the CRC model that would be used to describe the dynamics of a slower protein. CRC equations can also be easily modified to account for proteins that do not detach from DNA damage sites in the course of the experiments (i.e., no removal reaction). By means of the CRC model, we analyzed the kinetic behavior of all studied proteins using every time the minimal number of reactions (i.e., the simplest CRC equation) that could fully describe the dynamics of a particular DNA repair protein [9]. The DNArepairK database contains both the raw kinetics data and its respective CRC model for every protein included.

Notably, many DNA repair proteins included in DNArepairK exhibit complex kinetic behavior that cannot be sufficiently well described by a single CRC equation [9]. For some proteins, such as PARG, RFC4, RFC5, and FAN1, such complicated dynamics may be attributed to the fact that they are parts of several different protein complexes and/or participate in different processes during DNA repair, hence, they exhibit biphasic or even multiphase kinetic behavior that cannot be described by a single CRC equation (Figure 3b). Other proteins are not fully dissociated from complex DNA lesions in the end of the time lapse experiments, therefore, we assign them both “removable” and “non-removable” fractions to account for this behavior (Figure 3c). For several proteins the photobleaching during IR could not be fully compensated for during image analysis and their raw kinetics contain a “bleach” component which can be easily quantified and excluded from the overall kinetics (Figure 3d). In all these cases we managed to precisely describe the complex overall kinetic behavior by using a combination of two or more CRC equations to account for the different processes involved in these proteins’ recruitment and/or removal [9]. Importantly, following this approach, we managed to individually characterize the separate processes that govern these complex kinetic behaviors, providing information for their nature unattainable by other methods that can be readily accessed by the users of the DNArepairK database.

### 3.3. DNArepairK Provides a Comprehensive Overview of the Effects of Anticancer Drugs onto the Dynamics of the DNA Damage Response

Poly(ADP-ribose) polymerase 1 (PARP1) is an abundant nuclear protein, which, together with PARP2, acts as a DNA damage sensor by rapidly binding to single- and double-strand DNA breaks [24]. PARP1 binding to DNA lesions stimulates its enzymatic activity, and thereafter, PARP1 starts to synthesize and attach branched and strongly negative poly(ADP-ribose) (PAR) chains to nearby chromatin [25]. These PAR polymers formed at the site of the damage function as scaffolds for the recruitment and retention of downstream DNA repair factors. Hence, PARP1 recruitment to damaged DNA and its activation are pivotal in the first steps of DNA repair. PARP1/2 inhibitors (PARPis) are the first class of drugs acting by the principle of synthetic lethality that have been approved for clinical use for the treatment of homologous recombination (HR)-deficient types of cancer (mainly, BRCA1/2-deficient ovarian and breast cancers) [26]. Synthetic lethality is a novel paradigm in cancer treatment regimens that combines a cancer-inherent genetic mutation (such as BRCA1 or BRCA2 mutation) with the pharmacological inhibition of a specific molecular target (such as PARP1) [27,28]. These simultaneous perturbations are unviable and lead to profound cancer cell death. Curiously, PARPis are not only potent enzymatic inhibitors of PARylation, but they induce structural changes in PARP1, which prolong the time it spends bound to damaged DNA, an effect termed “PARP trapping” [29,30]. This dual mechanism of action of PARPis leads to a significant shuffle in the order of downstream events in the course of DNA repair, possibly diminishing its proficiency [9]. As of May 2021, there are four clinically approved PARPis that differ greatly in their inhibiting and trapping potencies, and a better understanding of the reasons behind these differences may lead to improved cancer patient treatment protocols [26].

**Figure 4:**
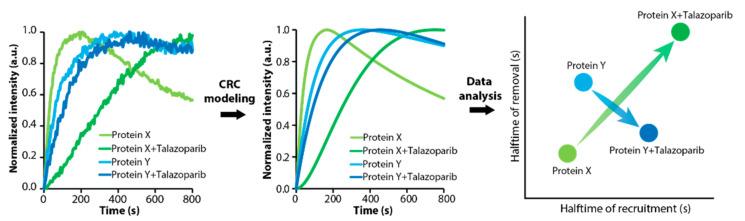
DNArepairK provides a comprehensive overview of the effects of talazoparib onto the dynamics of the DNA damage response. A comprehensive kinetics-based overview of the effects of talazoparib onto the dynamics of the DNA damage response can be obtained by measuring, CRC modeling, and comparing the kinetics of recruitment and removal of multiple DNA repair proteins in non-treated and talazoparib-treated cells.

As part of our study [9], we treated cells with talazoparib (BMN673), which is the most potently trapping PARPi discovered to date [10,30], and obtained the kinetics of the proteins of interest in the same experimental conditions, making the kinetic data readily comparable with the data obtained from non-treated cells (Figure 4). A detailed analysis and discussion of the findings is to be found elsewhere [9]. Briefly, we discovered that talazoparib treatment markedly altered the order of recruitment of multiple DNA repair proteins, leading to conditions that favor a more error-prone DNA repair process. In untreated cells, the recruitment of error-free DNA polymerases such as DNA polymerase delta (POLD) precedes by more than a minute the recruitment of error-prone translesion DNA polymerases such as DNA polymerases eta (POLH) and kappa (POLK), therefore providing the cells with a temporal window for executing proficient DNA synthesis and diminishing mutagenesis stemming from the repair process itself. PARPi treatment eliminates this temporal advantage for error-free DNA synthesis at complex DNA lesions and leads to the simultaneous recruitment of both types of polymerases preconditioning a more error-prone repair process [9]. In addition, we uncovered that the removal of PCNA from complex DNA lesions strongly correlates with the recruitment of the single-strand DNA-binding protein RPA1. This may be due to the fact that all PCNA-dependent repair events must be completed in the vicinity of a double-strand DNA break (DSB) before 5′>3′ DNA resection, a hallmark of the homologous recombination (HR) pathway, commences. PARPi disengages PCNA removal from RPA1 recruitment and RPA1 recruitment launches before the completion of PCNA-dependent repair processes and PCNA removal. This, in turn, could lead to severely reduced efficiency of homology search due to the presence of unrepaired DNA lesions in the vicinity of a DSB, consequently abrogating the efficacy of the homology-directed repair pathway [9]. This was the first and, to date, the only systematic investigation of the effects of an anticancer DNA repair targeting drug onto the overall dynamics of the repair process in living cells. Our approach provided a comprehensive and systematic understanding of the dynamic changes in the DNA repair process that accompany PARPi treatment and revealed unanticipated modes of action of PARPis. All these data are included and easily accessible as part of the DNArepairK database.

Importantly, the DNA damage response offers many more protein targets and other synthetic lethal interactions that could be employed in the clinic for treating various types of cancers [31]. Therefore, not surprisingly, a huge number of DNA repair targeting drugs are currently in development (ATMi, ATRi, DNA-PKi, etc.) [6]. The information in the DNArepairK database may inform and guide the development and evaluation of novel molecules and provide crucial insights into the normal dynamics of the DNA repair process and how it can be altered due to various pharmacological perturbations.

### 3.4. DNArepairK Database Structure and Accessibility

The DNArepairK database is an interactive, user-friendly, and freely accessible online database that does not pose restrictions of any kind on its users. For the creation of the database, the following technologies were used: WebGL API, JavaScript, HTML, and CSS. The WebGL API provides us with the ability to create 2D and 3D graphics through programmable shaders by using a canvas element adopted in the HTML5 standard. The technology is web-based and supported by modern web browsers such as Mozilla Firefox and Google Chrome. This makes the database independent of the underlying operating system. The visualization of experimental data, mathematical formulas, and user interactivity is achieved through a combination of HTML, CSS, and a rich set of JavaScript features.

**Figure 5:**
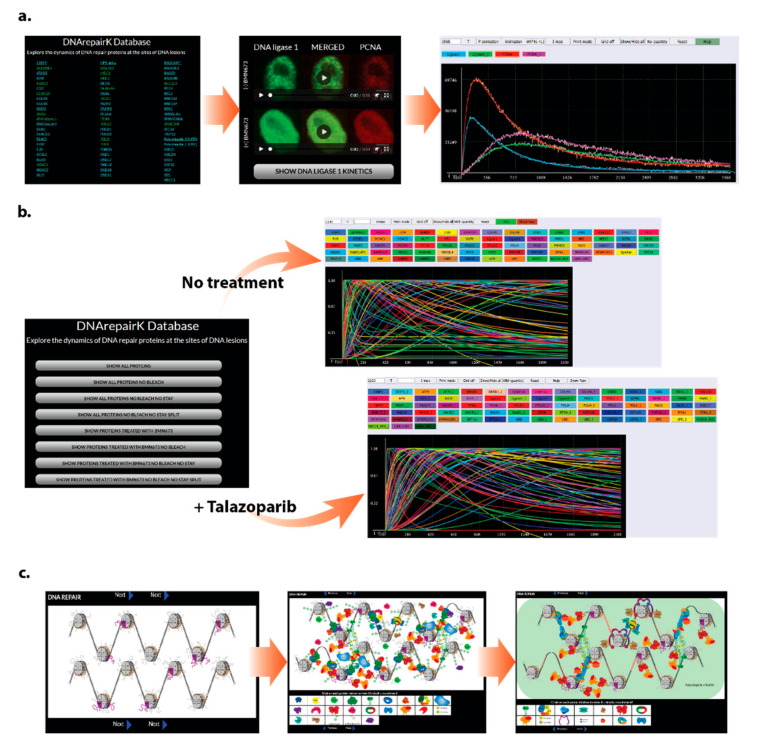
Structure of the DNArepairK database. (**a**) Kinetics of individual proteins. This part of the database contains the kinetics of all studied proteins accompanied by their CRC models and time lapse movies depicting their recruitment and removal at complex DNA damage sites in living cells. (**b**) Kinetics of all the proteins. The second section allows the user to comfortably compare the kinetics of all investigated proteins with and without talazoparib treatment. (**c**) Interactive pictures. The last section represents an interactive slideshow of the events in the course of DNA repair based on the kinetics data included in the DNArepairK database.

DNArepairK is composed of three interlinked sections, and the transition between them is quick and convenient for the user. The first section contains the individual kinetics of all 70 DNA repair proteins included in DNArepairK complemented with time lapse movies of the recruitment and removal of these proteins to DNA damage sites in living cells (Figure 5a). The second portion of the database offers the possibility for a systematic analysis of the kinetics of all proteins and investigating the global effects of talazoparib onto the dynamics of the DNA damage response (Figure 5b). The last section is composed of a series of interactive pictures that represent the chronology of the events in the course of complex DNA damage repair that is based on the kinetics data included in DNArepairK and the known properties and interactions between the proteins (Figure 5c).

#### 3.4.1. Kinetics of Individual Proteins

The first segment of DNArepairK encompasses the individual kinetics of all DNA repair proteins included in the database (Figure 5a). When users access any of the proteins, they will be provided with a time lapse movie depicting the localization and the dynamic behavior of the respective proteins at DNA damage sites obtained from time lapse live cell imaging experiments. The kinetics of most of the proteins were obtained both in non-treated and talazoparib-treated cells [9]. When this is the case, a movie showing the behavior of the respective protein in treated cells is also provided. The kinetics of many proteins (most notably, many PCNA-interacting proteins) were obtained from double cell lines expressing both the protein of interest labeled with EGFP and mouse PCNA (mPCNA) labeled with mCherry. In many of our experiments, we use the kinetic behavior of mPCNA, which is exactly the same as human PCNA, as a reference for the kinetics of the protein of interest [9]. When the kinetics of a protein is obtained in such a double cell line, an additional movie presenting the dynamics of mPCNA is also provided to the users. Immediately below the movie(s), a “Show kinetics” button is localized. By pressing this button, a new browser window will open that will display the raw kinetics data of the respective protein accompanied by the CRC model that best describes its kinetics. When the kinetics of a particular protein is obtained in both non-treated and talazoparib-treated cells and/or in the presence of mCherry-tagged PCNA, all these kinetics data will be available to the user as well. In this window, users can easily change both the timing on the x-axis and the total fluorescence intensity on the y-axis of the graph by recording the desired values in the boxes preceding the buttons “T” and “I-max”, respectively, and then pressing these buttons. The kinetics data can also be normalized by deleting the value in the “I-max” box and pressing the “No quantity” button. In many cases, the normalized kinetics is better suited for comparing the kinetics between different proteins or the kinetics of a single protein in different conditions. The button “Print mode” will change the graph background color from black to white, which is more suitable for printing, and pressing the “Grid off” button will eliminate the horizontal and vertical gridlines if this is preferred. Returning to a black background and presence of gridlines can be achieved by pressing the buttons “Normal mode” and “Grid on” that become active following the transition to printing mode and/or removing the gridlines. In order to facilitate data exploration, users can hide or show both the raw intensity data and the CRC models for the proteins of interest by pressing the “Show/Hide all” button with the right or the left mouse button, respectively. If users desire to remove the raw intensity data or the CRC model for a particular protein or condition (e.g., treatment vs. no treatment), they can achieve this by clicking on its respective button, situated just above the graph, following which, the edge or the button itself will change its color to white. This can be done for more than one protein at a time and can be reversed by clicking the button again. If something during data exploration goes wrong, the initial condition can be restored by simply pressing the “Reset” button. In order to facilitate the comparison between the behavior of a particular protein in non-treated and talazoparib-treated cells, users can visually observe an animation of the changes that occur in the kinetics of this protein by pressing either the “Animation” or “P animation” buttons. The “Animation” button will visualize the sequential changes in the kinetic constants that occur during the transition between the CRC models describing the kinetics of a protein in the absence or presence of talazoparib, thereby providing the users with a clear notion about the role of every single kinetic constant towards the complete kinetics of the respective protein. In contrast, the “P animation” button will display the parallel changes in the kinetic constants during this transition. Finally, the “Help” button will provide the user with a complete guide for the functions of the rest of the buttons if such is needed.

In addition, users are able to freely download (by clicking the “Download raw and fit data” button) an Excel spreadsheet for every protein, which includes the raw intensity data and its standard deviation for every time point, the CRC model that was used to theoretically describe the kinetics of the protein and its kinetic constants, and detailed data concerning the imaging and micro-irradiation conditions. This way, users are able to download our data, explore it, and compare it to their own, therefore greatly increasing the accessibility of the DNArepairK database. A “Protein ID” button promptly redirects users to the UniProt database page for the particular protein of interest, where they can obtain much more additional information about its functions and properties. Lastly, for every cell line included in the DNArepairK database, a BAC ID is provided that refers to the identity of the bacterial artificial chromosome that has been used for fluorescent protein labeling of the gene of interest.

This section of the database and the functionalities it offers is ideally suited to be exploited by the users for an in-depth analysis of the kinetic behavior of a particular DNA repair protein or a small group of proteins. In addition, users can meticulously investigate and compare the behavior of a particular protein(s) of interest in talazoparib-treated and non-treated cells, gaining insights into the effects of PARPi.

#### 3.4.2. Kinetics of All the Proteins

The second section of the DNArepairK database offers users a simultaneous global overview of the kinetics of all proteins included in the database both in non-treated and talazoparib-treated cells (Figure 5b). By exploring this part of the database, users can compare both the raw kinetics data and the CRC models for all the proteins in a normalized or non-normalized mode, gaining insights about the full chronology of events during DNA repair and the systematic effects of PARPi on DDR dynamics. By default, when accessing the data in this part of the database, users are provided initially only with the CRC mathematical models, since they are more convenient for making comparisons. If desired, users can load the raw kinetics data by simply pressing the “Show Raw” button above the graphics panel. Importantly, by switching off/on the tracks of particular proteins, it is possible to explore the chronology of a specific DNA repair pathway or event (e.g., comparing the kinetics and order of recruitment of proteins participating in double-strand DNA break repair by homologous recombination by showing only the tracks of RAD50, ATM, MDC1, RNF168, BARD1, RPA1, RAD17, RAD1, and RAD51). Such an accessibility option provides the users with an unbiased perspective towards the kinetics of a given protein and does not restrain DNA repair proteins to specific DNA repair mechanisms.

As discussed above, many proteins included in DNArepairK display complex kinetic behaviors that can be mathematically modeled only by means of a combination between two or more CRC equations. In this section of the database, users are able to inspect DNA repair proteins’ kinetics without the nonremovable fractions and without the bleach components. Importantly, when the proteins’ kinetics are complex due to diverse modes of protein recruitment to damage sites, users are provided with the individual CRC models describing these different modes (i.e., the composite kinetics are split into several distinct CRC curves for every mode of recruitment) named “fit1”, “fit2”, etc.

#### 3.4.3. Interactive Pictures

The last section of our database represents an interactive slideshow of pictures depicting the chronology of the main events during complex DNA damage repair, as revealed by our kinetic dataset (Figure 5c). The elaboration of this slideshow also relied on data concerning the specific functions and interactions between the proteins included in DNArepairK. By clicking on the individual protein molecules in the graphical list located immediately below every slide, users are promptly redirected to the kinetics of the respective DNA repair proteins, thereby greatly facilitating the exploration of this vast amount of information. To further increase the accessibility, users can zoom in or out with the help of the scroll button of the mouse on specific portions of the pictures.

#### 3.4.4. MolDViewer—An Application to Import Custom Kinetics Data and Mathematical Models

MolDViewer is a multiplatform, web-based, open-source application, which provides a functional and user-friendly environment for comparing the experimentally obtained kinetics of diverse cellular processes and their mathematical models. MolDViewer comprises a comprehensive set of features required for kinetics data visualization and a convenient way for generating online interactive kinetics-based databases such as DNArepairK. By using MolDViewer, end users have the possibility to interactively place their own custom mathematical models and experimentally obtained data by uploading a single file for each data type by pressing the “Fit data” and “Raw data” buttons, respectively. The kinetic constants characterizing the employed mathematical models can be visualized/hid by pressing the “Show consts/Hide consts” button, and while shown, they can be modified by the user in order to explore the contribution of every single constant towards the overall kinetics. The respective changes in the overall kinetics following the changes in the kinetic constants are readily visualized in the graphics panel. By pressing the “Show sliders” button, users are able to set a defined interval for every kinetic constant and, then, by pressing the “Animation” button to visualize the changes in the overall kinetics of the protein in a user-defined number of steps. In addition to this, users may define the names and colors of the graphs, normalize the data, hide and show both the mathematical model and the raw data for every single protein or of all the visualized proteins at once, and set the grid and background colors of the graphics, as well as to animate the depicted processes in various ways to cover the practical use cases when analyzing the kinetics of cellular processes. Step-by-step instructions for using MolDViewer (“MolDViewer Tutorial” document file) and ready-to-upload equations and raw data (“Fit and Raw Data” folder) for all proteins and conditions included in the DNArepairK database are provided as Supplementary Materials. A training dataset for MolDViewer is freely accessible at github.com (link provided in the Data Availability section at the end of the manuscript).

MolDViewer is a web-based solution combined with a file upload capability that allows the scientists using it to easily share and compare ideas and results. Once the end users reach their final results with the chosen mathematical model and raw dataset, they can use the permanent data visualization version of MolDViewer to show their own results in a web-hosted manner, as well as to add a capability for free browsing between the experimentally obtained kinetics, the mathematical models describing the data, and the source image files from which the kinetic data were obtained, e.g., time lapse microscopy movies. MolDViewer has the potential to facilitate and simplify the exploration of the kinetics of a multitude of cellular processes, as demonstrated for the highly complex dynamics of the DDR.

## 4. Discussion

Macromolecular interactions are fundamental to cell physiology [32]. Over the past decades, the number of protein–protein, protein–DNA, and protein–RNA interactions identified has increased significantly, and several databases have been created to annotate these interactions [33]. However, an in-depth understanding of the complex cellular processes requires comprehensive knowledge about the kinetics of these processes in living cells and how the usual dynamics of a particular process can be altered due to various internal or external perturbations. With the advancement of live cell imaging technologies, such a knowledge is becoming more and more accessible and fuels the rapid development of novel treatments for numerous maladies [34].

The DNArepairK database presented here is unique in that it provides an unparalleled overview of the dynamics of the DNA damage response, since it encompasses the high-resolution kinetics of recruitment and removal of 70 different DNA repair proteins to UV laser-induced DNA damage sites in living cells. This vast set includes proteins contributing to all events occurring in the course of DNA repair, and all the kinetics have been obtained under identical experimental conditions, making the data readily comparable. The kinetics for every single protein is mathematically modeled by means of the Consecutive Reactions Chain (CRC) model, providing the users of the database with even more insights into the complex kinetic behavior of many DNA repair proteins. DNArepairK is interactive, easy to navigate, and drastically simplifies the comparisons between the kinetics of different DNA repair proteins, thereby facilitating the exploration of the highly complex dynamics of the DNA damage response. Moreover, the MolDViewer platform can be employed by the users to upload their own data and apply the CRC or other mathematical models to theoretically describe their data.

The accumulation of lesions in DNA is directly linked to cell transformation and tumorigenesis [3]. Moreover, many different types of cancers carry disabling mutations in prominent DNA repair genes [35]. Therefore, deeper knowledge of the DNA damage response will undoubtedly prove to be pivotal in the creation of novel, more rational, and effective anticancer therapies. Several PARP1/2 inhibitors that target HR-deficient types of cancer have already entered the clinic with many more PARPis in various stages of development [36]. While the direct mechanisms of action of these drugs have been fairly well explored, their systematic effects onto the overall dynamics of the DNA repair process were not examined until recently. The exclusive dataset shared in DNArepairK sheds light on the complex changes and dramatic alterations in the dynamics of the DNA damage response that accompany PARPi treatment in living cells. Such information may be of great value in developing and evaluating novel PARPis, as well as in repurposing already approved PARPis for the treatment of different types of cancer. DNArepairK provides its users with easy navigation and the means to explore the changes in the dynamics of particular DNA repair proteins, as well as the changes in the overall dynamics of the DNA damage response following talazoparib treatment.

We expect that the DNArepairK database will be duly updated with new kinetics of other DNA repair proteins and/or treatment conditions obtained by employing the stable transgenic HeLa Kyoto cell lines that our research group has adopted as a model system. In addition, DNArepairK and the DNA repair community could hugely benefit if similar kinetic datasets obtained by other research groups are included as part of the database—notably, kinetic data from diverse biological systems and/or distinct microscopy and micro-irradiation setups. Such external data can be readily incorporated in the current structure of the database and could shed light onto the contextual nature of the DNA damage response. In conclusion, we believe that the DNArepairK database will be beneficial for many scientists working in the field of DNA repair, providing them with a unique kinetics-based dataset illuminating the complicated and intertwined chain of events arising after DNA damage infliction in living cells. A better understanding of the dynamics of the DNA repair process and how it is modified in certain conditions may prove to be pivotal in the creation of novel, more efficient, and sparing cancer treatments adapted to the specific genetic backgrounds of the patients, a keystone in personalized medicine. Therefore, we anticipate that DNArepairK will also be of great value to scientists developing novel DNA repair targeting anticancer drugs and to cancer biologists and oncologists investigating the multifaceted connections between tumorigenesis and the DNA damage response.

## Data Availability

The DNArepairK database is freely accessible and can be explored at http://dnarepair.bas.bg/index.php/dnarepairk/ (accessed on 6 September 2021) without any restrictions. MolDViewer can be accessed at: http://dnarepair.bas.bg/index.php/moldviewer (accessed on 6 September 2021), and a training dataset and step-by-step instructions for using all the features of MolDViewer can be found at: https://github.com/yordanbabukov/MolDViewer (accessed on 6 September 2021).

## Supplementary Materials

The following are available online at https://www.mdpi.com/article/10.3390/biomedicines9091238/s1: the “MolDViewer Tutorial” document file contains a comprehensive description of the features of the MolDViewer application and how users can upload and explore their own kinetics data in MolDViewer. The “Fit and Raw Data” folder contains all the experimental kinetics data that we have obtained and included as part of the DNArepairK database and the respective CRC models for the kinetic curves in a ready-to-upload format for visualization and comparison in MolDViewer.
