# Supplementary material for: DNArepairK: An Interactive Database for Exploring the Impact of Anticancer Drugs onto the Dynamics of DNA Repair Proteins
Source: Biomedicines. 2021 Sep 16;9(9):1238. doi: 10.3390/biomedicines9091238 (PMC8470695; doi:10.3390/biomedicines9091238)
Supplement: Supplementary file 1 [file biomedicines-09-01238-s001.zip › MolDViewer Tutorial.pdf]

Supplementary Material

1. Access MolDViewer and Training Materials

1.1. Access

1. Start MolDViewer by opening the following link https://dnarepair.bas.bg/MolDViewer.html
2. Expected result:

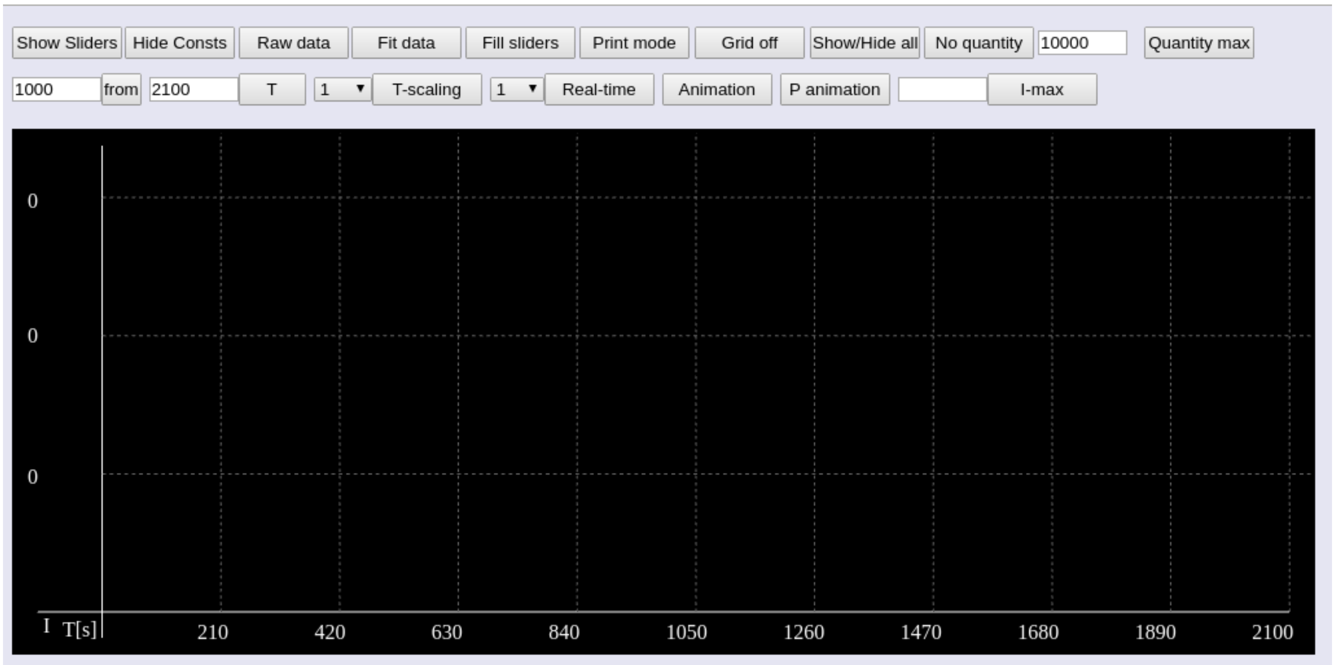

1.2. Training Materials

1. Location: https://github.com/yordanbabukov/MolDViewer
2. The training materials can be browsed either online or be downloaded locally on your PC
   - Select “Clone or download”
   - From the drop down menu choose “Download ZIP”

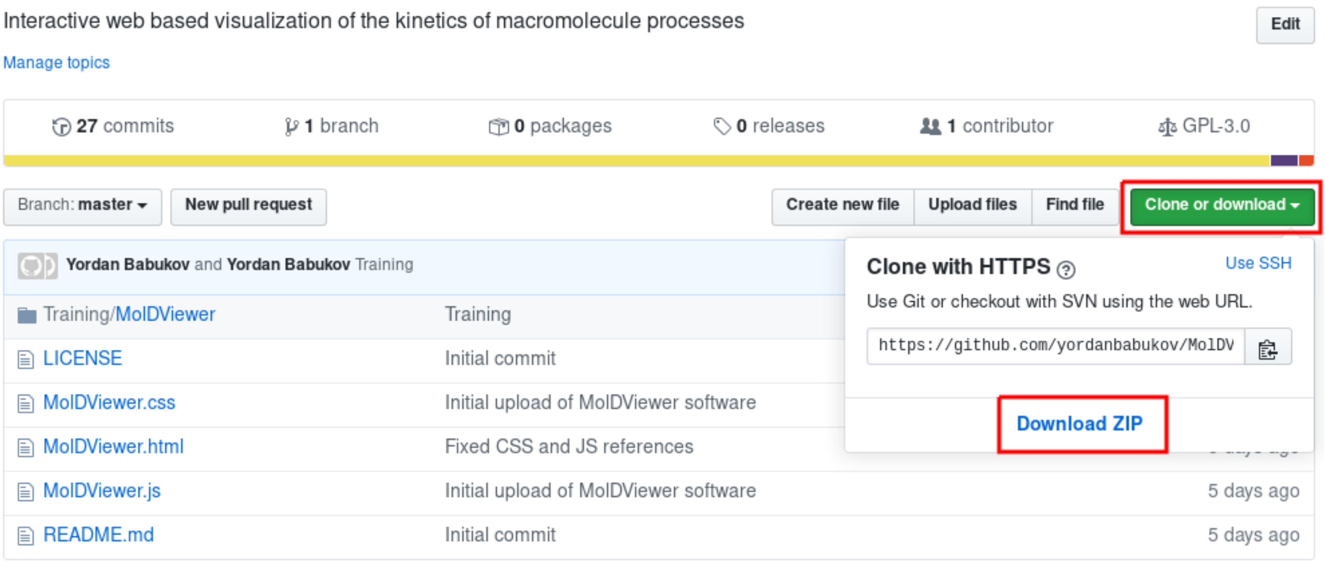

2. Fit Data, Color Handling and Constants

2. 1.“Fit Data” Button

This button will prompt you to upload a file. Content file format is discussed later.

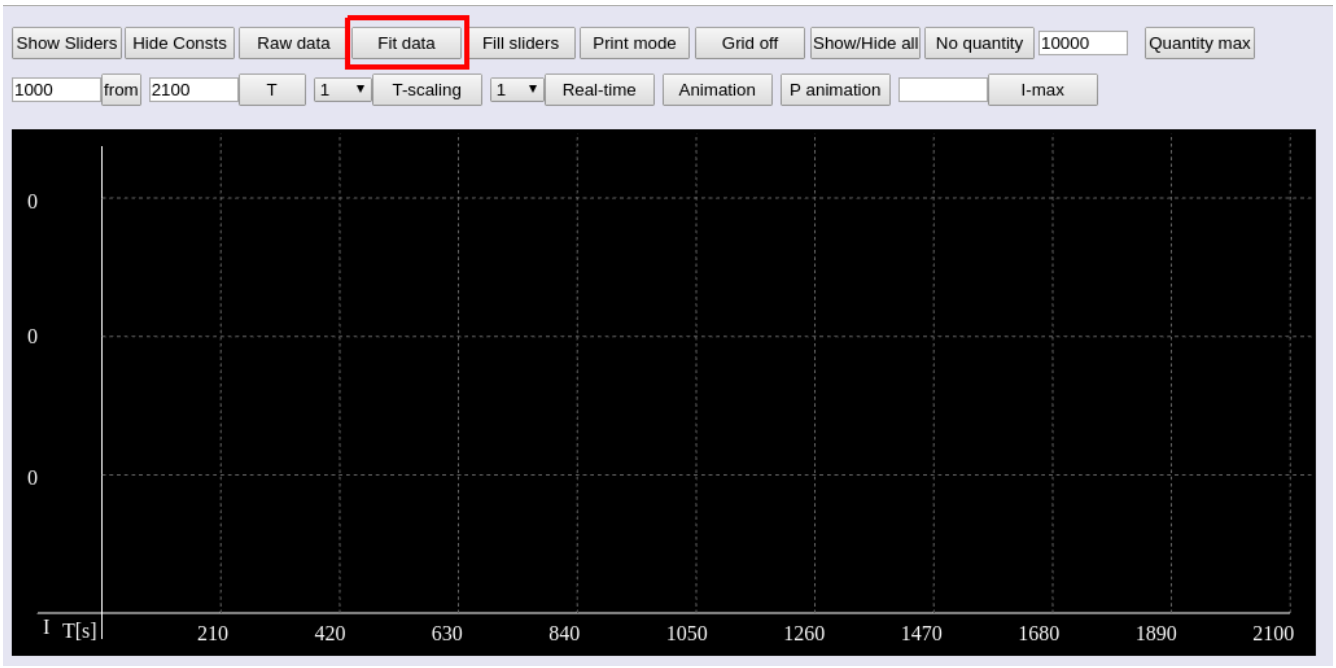

Upload file “**RPA.txt**” located in directory **2_Fit_Data**. The results should be the following:

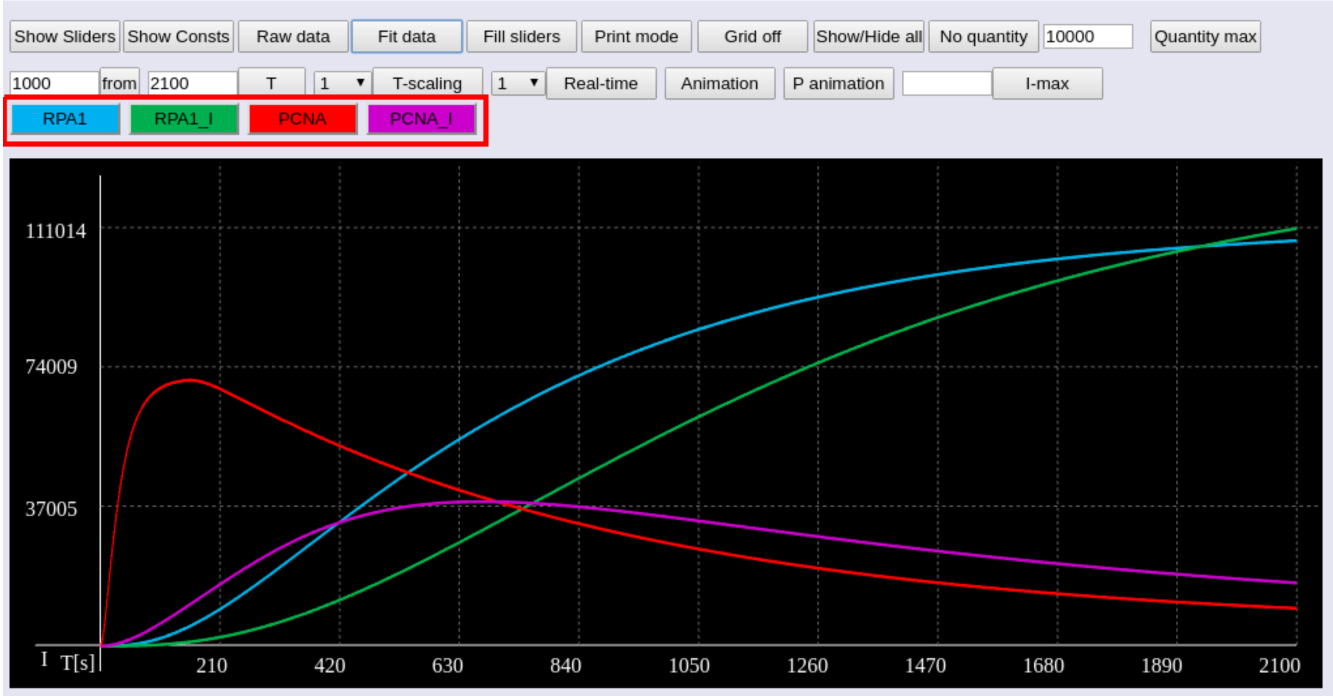

The **X axis** shows the total fluorescence intensity over time in seconds during the DNA repair process.

The **Y axis** depicts the total fluorescence intensity of recruited DNA repair protein molecules.

Additionally, buttons with the names and colors of the plotted graphs appear. For example, the red plotted graph is the modelled kinetics of PCNA and RPA1 is the blue one.

2.2."Show Consts" Button

This button displays the array of kinetic constants used in the equations to generate the graphs as well as their current values. Each value can be interactively modified from the end user as well as each color from the rightmost button on each row.

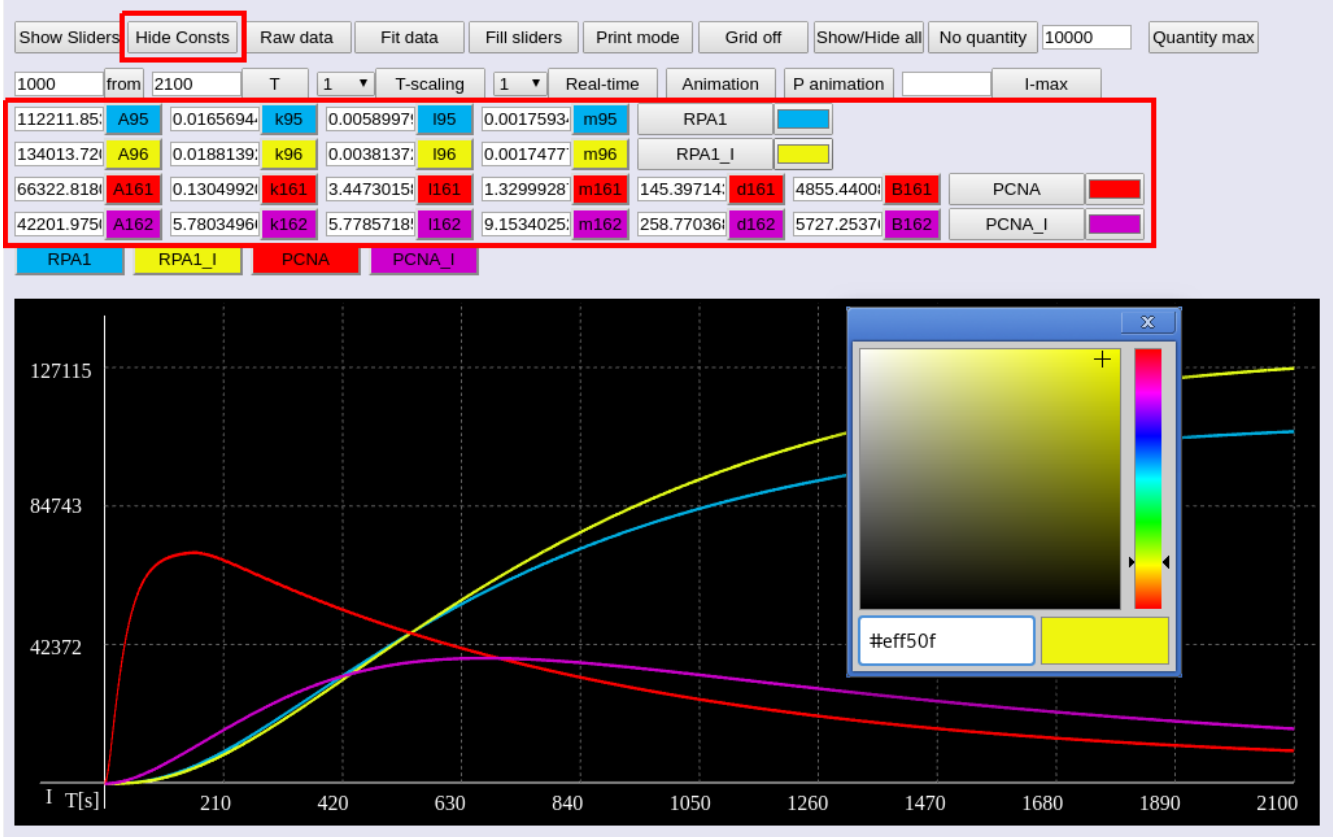

Here we changed RPA1_I constants' values as follows:

A96: 132013.720034804 → 134013.720034804

k96: 0.00381392331001804 → 0.01881392331001804

l96: not changed

m96: 0.00124777264634967 → 0.00174777264634967

Value changes can be applied by both pressing the "Enter" key or by a left mouse click on the button with the name of the constant.

As a result, the RPA1_I chart changed and the displayed intensity scaled automatically to provide once again fully scaled graphics on the canvas.

2.3. Color Handling

The graphics of RPA1 and RPA1_I are now too close to each other so users may decide to change the color of RPA1_I from RPA1_I's color button. In this way users can easily distinguish proteins with similar behavior.

2.3.". Hide Consts" Button

When modifying colors and values users can hide the expanded information by pressing the **"Hide consts"** button showed at the picture above.

3. Raw Data Upload, Handling X and Y Axes

Before uploading the raw data, a fit data upload is required*.

For this training the fit data set is "**3_Fit_Data/Ligase4_PCNA.txt**". Users can upload it by using the materials covered in the previous section of the training.

*To upload a new dataset, a refresh of the web page is required.

3.1.". Raw Data" Button

This button will prompt for a file upload. Contents file format is discussed later.

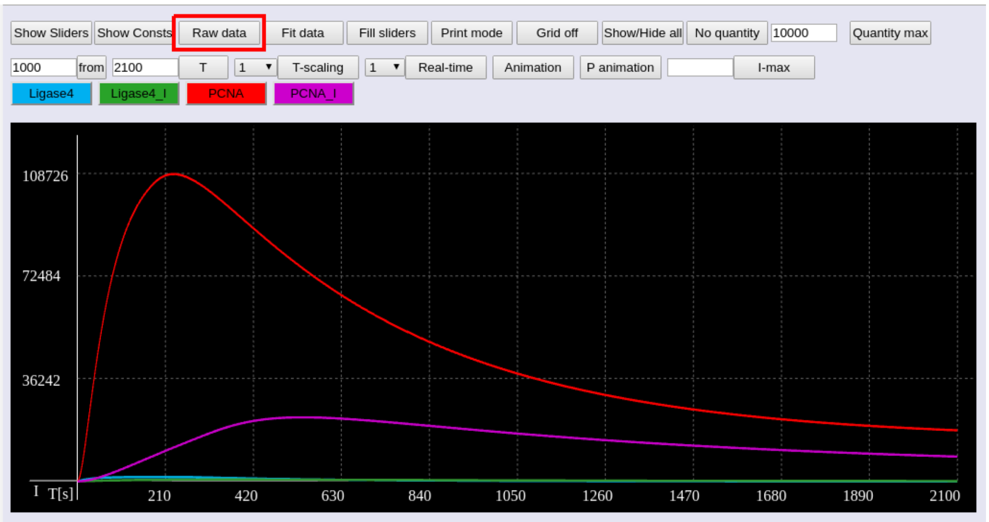

Upload all the files located at folder "**3_Raw_Data**" at once. The result should be the following:

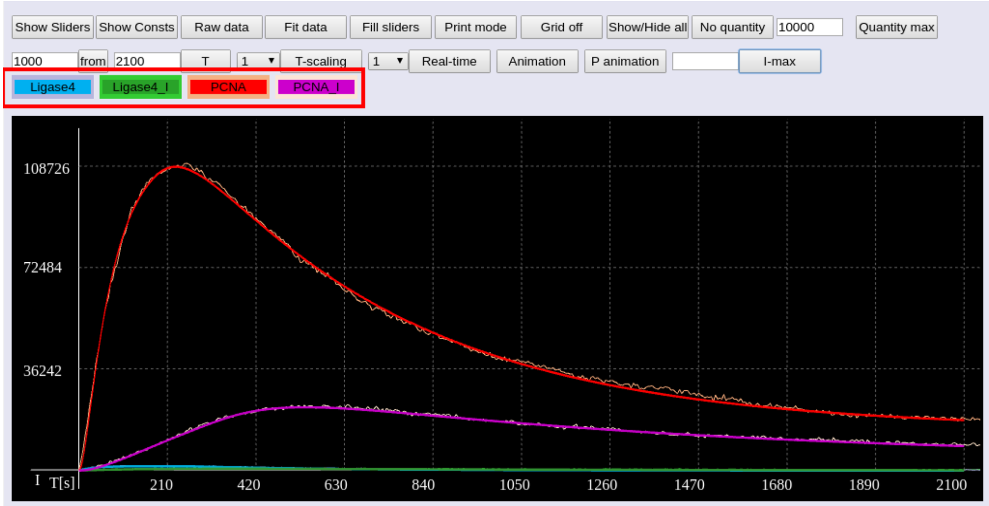

The raw datasets associated with a fit data equation will be shown on both the plotted graph itself as well as the border around the protein button. Here the raw dataset associated with PCNA (red graph) is with an orange color which could be seen on the plotted graph and as a border color around the PCNA button.

3.2. "I-max" Button

When at least one kinetic track has a high intensity value, the low intensity ones are hard to see. There is an **I-max button** to solve this issue. Add value 1500 and press either the "Enter" key or click on the **I-max button** to apply the change. The result should be the following:

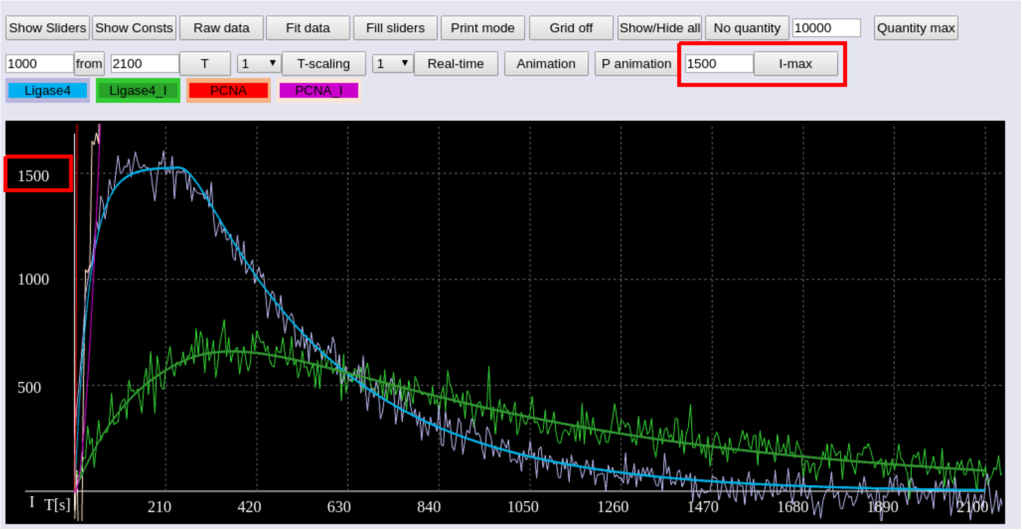

3.3.". No Quantity" Button

In real life there exists another use case when users need to visualize simultaneously both high and low intensity protein behaviors. To handle such situations there is a **"No quantity" button** that normalizes the graphs. To use the **"No quantity"** capability, users have to remove the I-max value and press either the "Enter" key or the **"I-max" button**. Now the intensity factor is ignored and all displayed fit and raw data are normalized. The result should be the following:

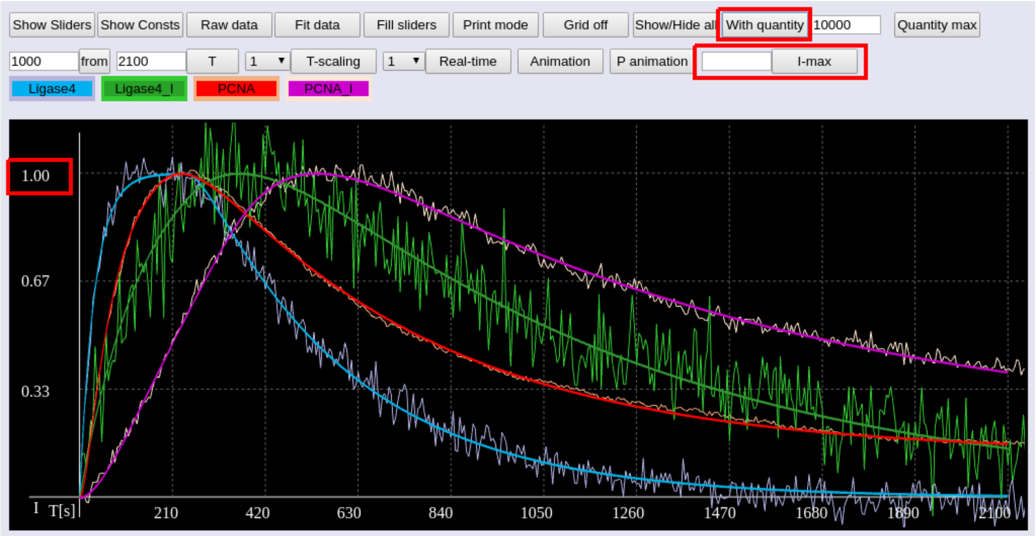

3.4." T" Button

During the systematic study of proteins there are usually situations when some proteins exhibit fast recruitment and detachment speeds compared to others. For example, on the picture above it is hard for the users to explore the proteins' behavior in the beginning of the kinetic curves. To handle these use-cases users can employ the **"T" button**. Add value 600 at the field on the left from the **"T" button** and press either thr "Enter" key or click on the **"T" button** itself. The result should be the following:

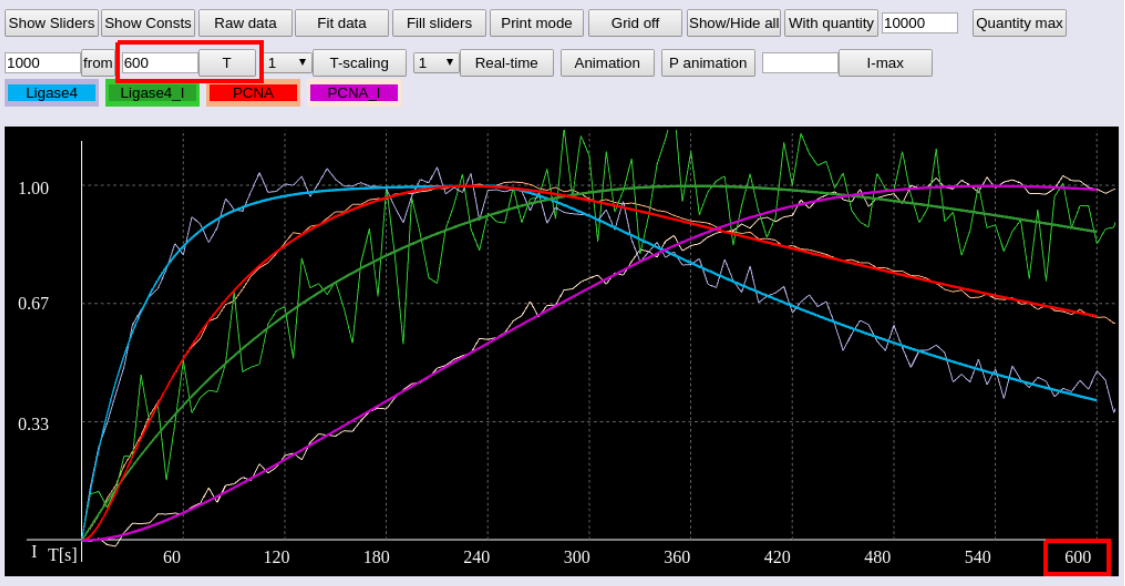

4. Show and Hide Capabilities. Paper Prettifying

4.1. Preparations

Upload the raw and fit datasets from the previous training.

4.2. Show/Hide Individual Graphs

Sometimes users will need to visualize fewer kinetic curves so they have a clearer picture. By clicking with the left mouse button on the proteins' names users can hide the desired fit data and with the right mouse button - the corresponding raw data. Once a fit curve is hidden the protein's button becomes white colored. When a raw data graph is hidden the border of the protein's button becomes white colored. For a demonstration purpose we will hide the fit data of Ligase4, the raw data of PCNA, PCNA_I and the fit and raw data of Ligase4_I. The result should be the following:

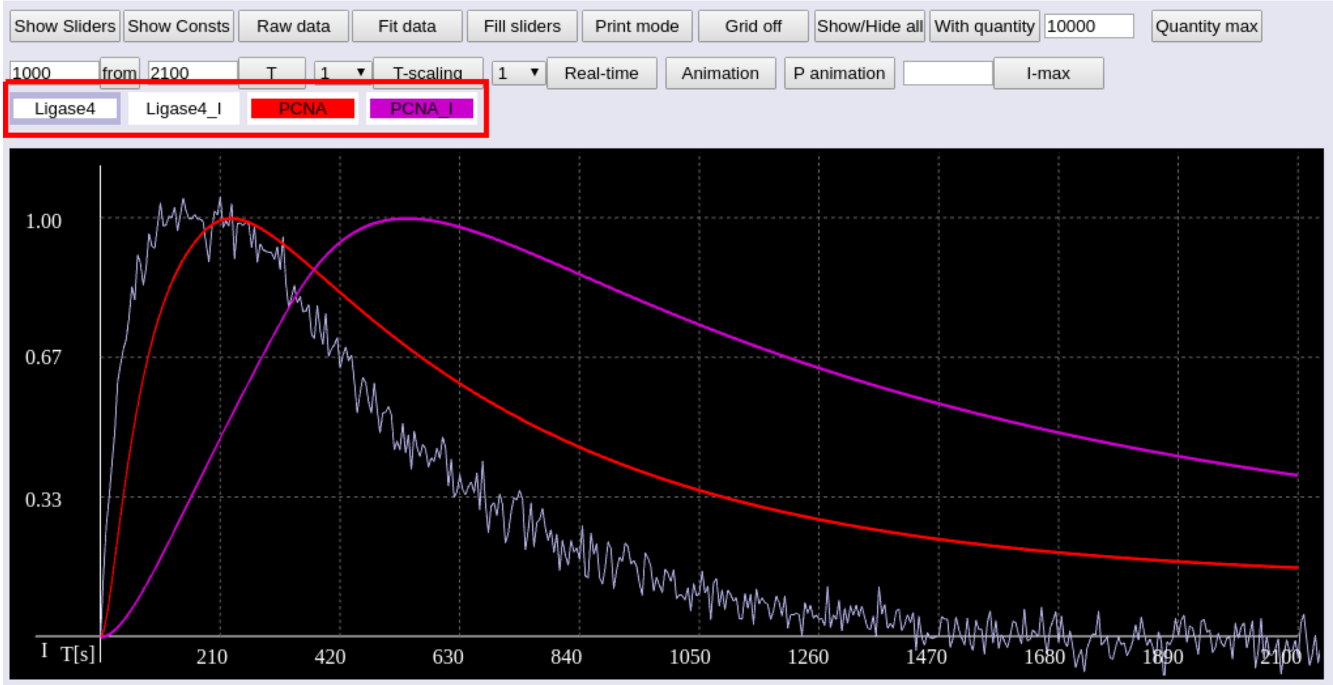

4.3. “Show/Hide All” Button

This functionality allows the user to show or hide all plotted data based on the specific use-case. By clicking with the left mouse button you can hide or show all the fit data graphs and with the right mouse button users can do the same for the raw data. We will now hide all raw data by clicking with the right mouse button on the “Show/Hide all” option. The result should be the following:

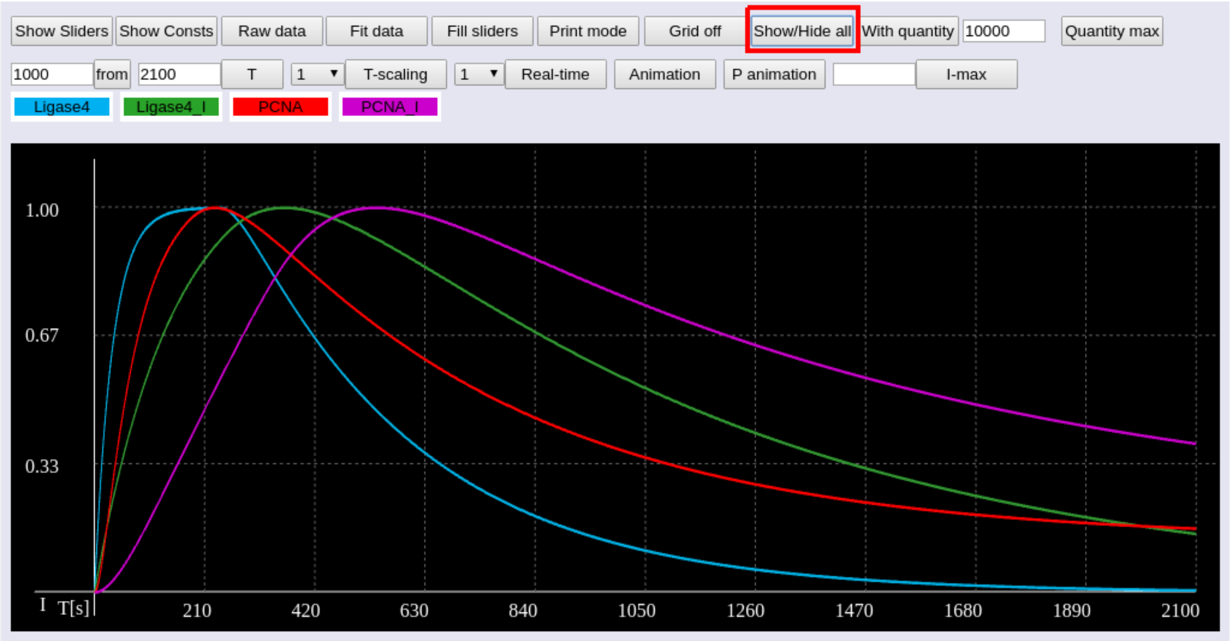

The same could be done for the fit data set. Click with the right mouse button to display the raw data again and after that click with the left mouse button to hide all fit data. The result should be:

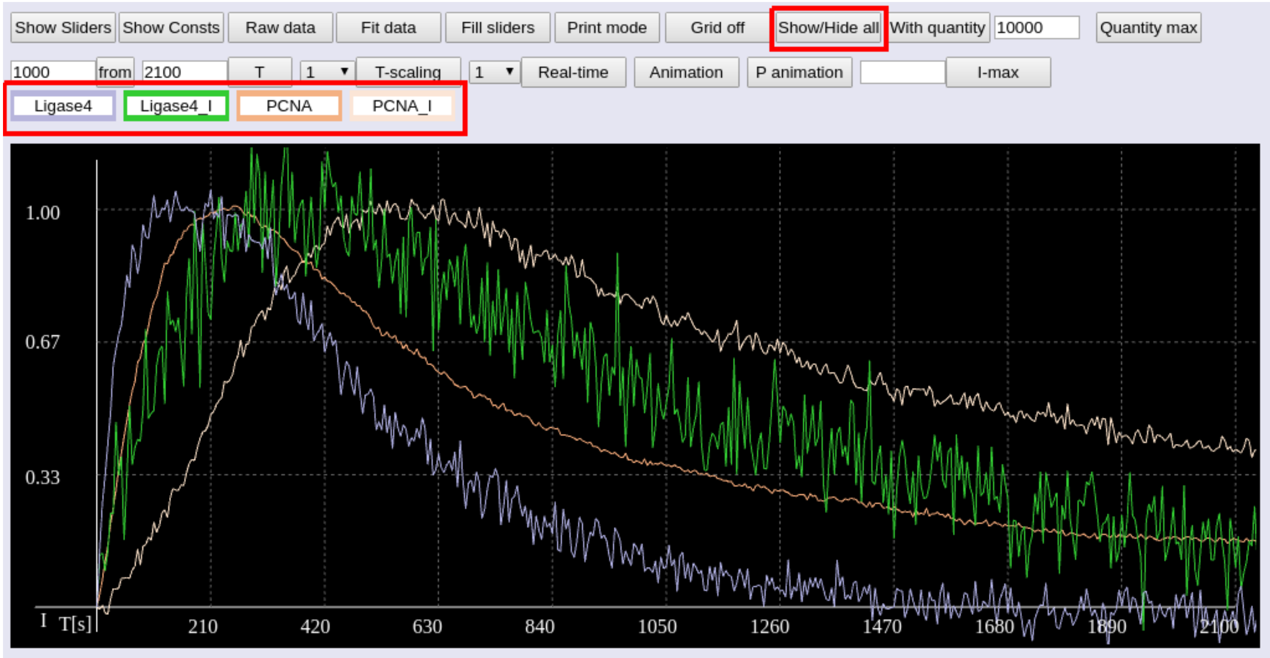

4.4. “Print Mode/Normal Mode” Button

The default background color of the canvas is black for improved color contrast. In order to be compatible with papers which have requirement for the background color to be white there is an option named **"Print mode"**:

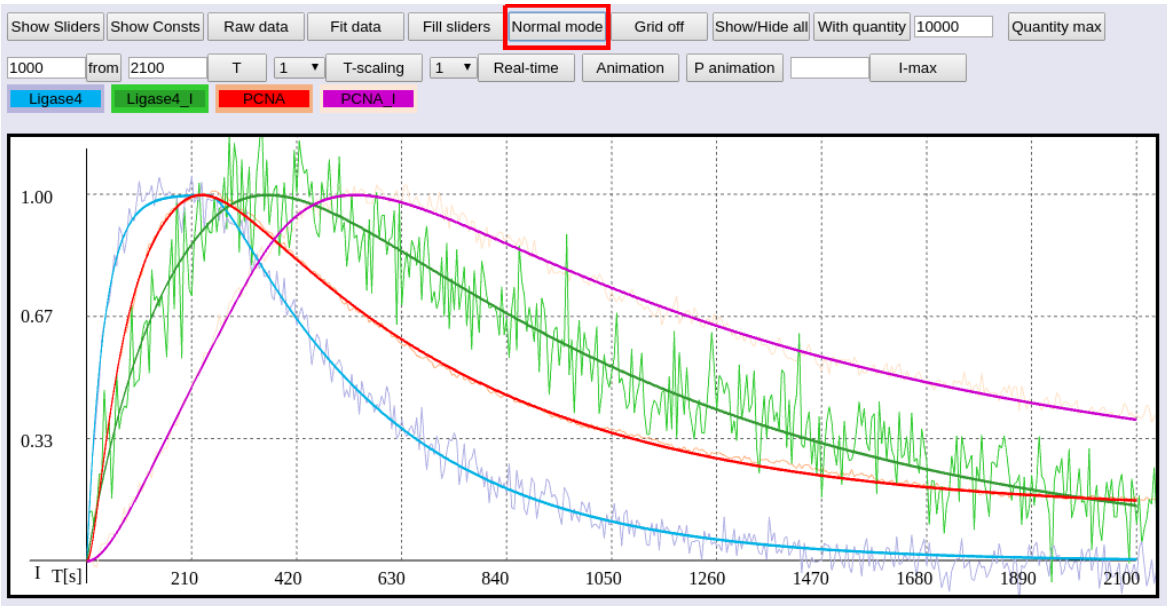

By pressing **"Normal mode"** the background color will turn black again.

4.5. "Grid On/Off" Button

Again, for paper compatibility there is an option to remove the stripped grid:

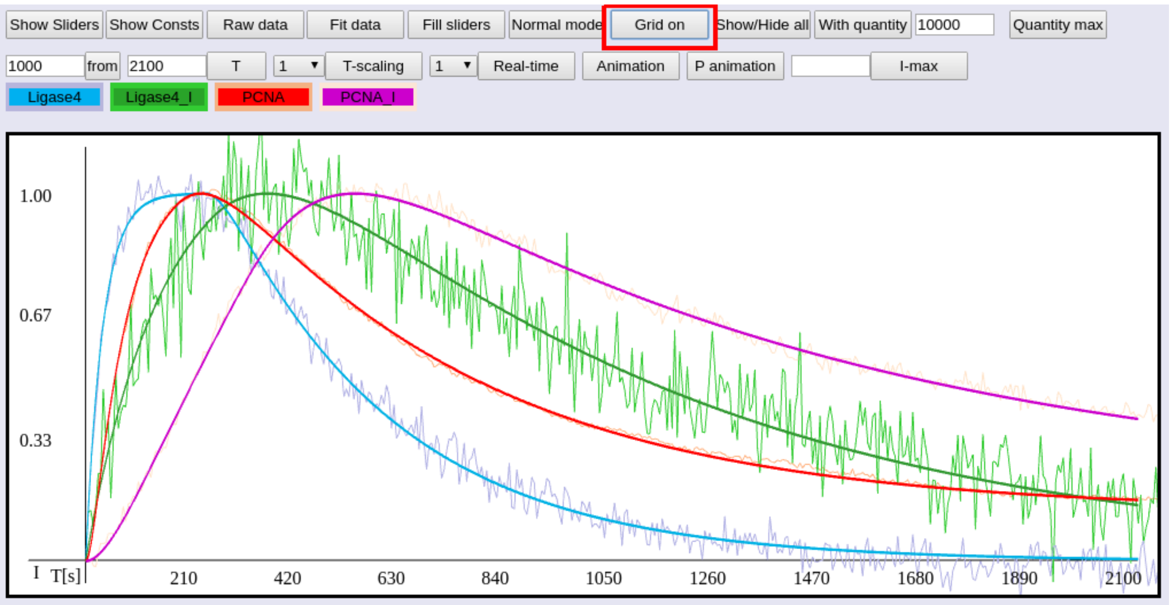

5. File Formats. Raw, Fit and Sliders Data

5.1. Raw Data File Format

Line 1: Name Color
Line 2: Moment_of_Measurement Measured_Value
...
Line n: Moment_of_Measurement Measured_Value

5.2. Fit Data File Format

Supports both equations with and without treatment conditions.

Line 1: Total number of equations
Line 2: Total number of sliders
Line 3: Equation without condition
Line 4: Equation color
Line 5: Constants and values [separator ';']
Line 6: Name of the equation
Repeat steps 3 to 6 for each subsequent equation
…
Line n-5: Equation with condition [before the condition is fulfilled]
Line n-4: Equation color
Line n-3: Constants and values [separator ';']
Line n-2: Name of the equation
Line n-1: Conditional parameter and value
Line n: Equation with condition [after the condition is fulfilled]
*Each equation should have dependent variable "t"

5.3. Sliders Data File Format

Line 1: Name_of_Variable Start_Value End_Value Step
Line 2: Name_of_Variable Start_Value End_Value Step
…
Line n: Name_of_Variable Start_Value End_Value Step

5.4. Examples

Filled files for reference:
Raw data files directory: **"Training/MolDViewer/6_Raw_Data/"**
Fit data files directory: "Training/MolDViewer/6_Fit_Data/"
Sliders data files directory: "Training/MolDViewer/6_Fill_Sliders/"

5.5. Raw Data File

```
PCNA    f4b183
0   0
5   3471.478802
10  7507.813771
15  12802.00737
```

5.6. Fit Data File

Sliders data file

```
2
10
 A32 1602762.22833435 1002762.22833435 20
 k32 0.0109153621785278 0.0009153621785278 20
 l32 0.00632900303522927 0.12632900303522927 20
 m32 0.000172106320547358 0.100172106320547358 20
 A47 1129415.57608335 909415.57608335 20
 k47 0.0463974576989205 0.0063974576989205 20
 n47 0.00003244025714157 0.03003244025714156 20
 l47 0.0464078720355239 0.08640787 20
 m47 0.00601337220436062 0.11601337220436062 20
Ligase4_I
if:t>=d43
A43*((k43/(k43-l43))*Math.exp(l43*(d43-t))-(l43/(k43-l43))*Math.exp(k43*(d43-t))-Math.exp(-k43[illegible]
```

MolDViewer necessitates the upload of a Fit data .txt file before uploading Raw data files as described above. Therefore, if users want to visualize only raw data, they need to prepare a .txt Fit file with all kinetic constants equaling zero. For the users to be able to see the raw data, they will need to choose an appropriate value in the I-max box in order to rescale the Y axis. If users want to visualize several raw data files at the same they, the corresponding number of equations in the Fit data file should be included (i.e. 5 equations in the Fit data .txt file for 5 different Raw data files).
